# Comparative Analysis of Perioperative Outcomes, Complications, and Early Renal Function After No-Touch Adaptive Versus Conventional Robot-Assisted Partial Nephrectomy for Clinically Localized Renal Tumours

**DOI:** 10.3390/jcm15166233

**Published:** 2026-08-12

**Authors:** Gianluca Giannarini, Eleonora Bovolenta, Giuliana Lista, Luca Di Gianfrancesco, Jeanlou Collavino, Clara Ermacora, Carmine Franzese, Afrovita Kungulli, Davide Minardi, Marco Rinaldi, Emma Segalla, Sasa Sekulovic, Fabio Traunero, Matteo Alberto Maniero, Francesco Claps, Maria Abbinante, Gioacchino De Giorgi, Antonio Amodeo, Angelo Porreca, Alessandro Crestani

**Affiliations:** 1 Urology Unit, Santa Maria della Misericordia University Hospital, 33100 Udine, Italy; eleonora.bovolenta@asufc.sanita.fvg.it (E.B.); jeanlou.collavino@asufc.sanita.fvg.it (J.C.); clara.ermacora@asufc.sanita.fvg.it (C.E.); carmine.franzese@asufc.sanita.fvg.it (C.F.); afrovita.kungulli@asufc.sanita.fvg.it (A.K.); davideminardi.15@gmail.com (D.M.); marco.rinaldi@asufc.sanita.fvg.it (M.R.); emma.segalla@asufc.sanita.fvg.it (E.S.); sasa.sekulovic@asufc.sanita.fvg.it (S.S.); fabio.traunero@asufc.sanita.fvg.it (F.T.); maria.abbinante@asufc.sanita.fvg.it (M.A.); gioacchino.degiorgi@asufc.sanita.fvg.it (G.D.G.); alessandro.crestani@asufc.sanita.fvg.it (A.C.); 2 Oncological Urology Unit, Veneto Institute of Oncology IOV–IRCCS, 35128 Padua, Italy; giuliana.lista@iov.veneto.it (G.L.); francesco.claps@iov.veneto.it (F.C.); antonio.amodeo@iov.veneto.it (A.A.); 3 Urology Unit, Humanitas Gavazzeni, 24125 Bergamo, Italy; luca.digianfrancesco@gavazzeni.it (L.D.G.); angelo.porreca@hunimed.eu (A.P.); 4 Department of Medicine (DMED), University of Udine, 33100 Udine, Italy; matteo.alberto.maniero@gmail.com; 5 Department of Biomedical Sciences, Humanitas University, 20072 Pieve Emanuele, Italy

**Keywords:** kidney cancer, renal cell carcinoma, robotic surgery, partial nephrectomy, renal function, enucleation, renorrhaphy, ischemia, complications

## Abstract

**Background**: Robot-assisted partial nephrectomy (RAPN) has become the preferred surgical approach for localized renal tumours, yet the optimal surgical technique remains uncertain. Ongoing refinements aim to reduce morbidity while maximizing renal function preservation. In this study, we compared perioperative outcomes, postoperative complications, and early renal function between the no-touch adaptive RAPN technique, designed to minimize vascular and parenchymal trauma, and the conventional approach. **Methods**: We retrospectively analyzed 387 consecutive patients undergoing RAPN for localized renal tumours between June 2017 and February 2026 at a single tertiary referral centre. Patients were treated with either the no-touch adaptive (*n* = 178) or conventional (*n* = 209) technique. The no-touch approach consisted of sutureless off-clamp simple tumour enucleation with incremental haemostasis and on-demand conversion to arterial clamping, tumour enucleoresection, or renorrhaphy when required. The conventional technique consisted of an on-clamp minimal enucleoresection with double-layer renorrhaphy. Completion rate of a fully no-touch procedure was assessed in the study group, and perioperative outcomes, 90-day complications, and renal function at 3 months were compared between the groups. Exploratory multivariable logistic regression identified predictors of postoperative complications. Absolute and relative changes in estimated glomerular filtration rate (eGFR) from baseline were analyzed, and a linear regression model adjusted for baseline eGFR was used to compare postoperative renal function between the groups. **Results**: Among the 175 evaluable patients after excluding three immediate conversions to radical nephrectomy before tumour excision, a fully no-touch procedure was completed in 153 (87.4%). Compared with conventional RAPN, the no-touch group had a significantly shorter hospital stay (3 vs. 5 days, *p* < 0.01), lower 90-day readmission rate (0% vs. 4.3%, *p* = 0.01), fewer overall complications (16.3% vs. 40.7%, *p* < 0.01), and fewer major complications (3.9% vs. 11%, *p* = 0.02), with no cases of delayed vascular bleeding. Low tumour complexity and the no-touch technique independently predicted a lower risk of postoperative complications. The median decline in eGFR from baseline to 3 months was significantly smaller in the no-touch group, which also showed a lower rate of clinically meaningful renal function deterioration (eGFR decline ≥ 30%; 1.1% vs. 5.7%, *p* = 0.03). After adjustment for baseline eGFR, the no-touch adaptive technique was associated with a 5.4 mL/min/1.73 m^2^ higher 3-month eGFR than the conventional technique (95% CI 3.6–7.2; *p* < 0.001). **Conclusions**: Within the limitations of this retrospective comparative study, the no-touch adaptive technique for RAPN was associated with lower perioperative morbidity and better preservation of early renal function than the conventional approach. These findings support further prospective multicentre studies to determine the reproducibility, durability, and clinical relevance of this surgical strategy.

## 1. Introduction

With the currently expanding therapeutic armamentarium, treatment decision-making for patients with clinically localized renal tumours has become increasingly complex, and requires a shared discussion that considers patient-, renal function-, tumour-, and provider-related factors, all of which may directly or indirectly influence key decisional points [1].

For patients opting for surgery, shared decision-making about the risk-benefit trade-offs between partial nephrectomy (PN) and radical nephrectomy (RN) is crucial, particularly for large or anatomically complex tumours where pursuing PN may carry higher perioperative and potentially oncological risks [2,3]. According to the latest guidelines, PN is recommended in all patients with T1 tumours, and in selected patients with T2 tumours and a solitary kidney or chronic kidney disease if technically feasible [4].

In recent years, robot-assisted PN (RAPN) has progressively replaced open partial nephrectomy as the preferred surgical approach, owing to advantages such as reduced blood loss, lower perioperative morbidity, and faster convalescence, while maintaining equivalent oncological outcomes [5,6]. This shift in practice has been accompanied by an increasing focus on optimizing functional outcomes, including reduction in surgical morbidity, preservation of renal function, and improvement in patient quality of life, largely facilitated by ongoing refinements in surgical technique.

Despite more than two decades of clinical use, RAPN still lacks a universally standardized surgical protocol. Key technical steps, including renal artery management, tumour excision technique, and renal parenchymal reconstruction, may each significantly affect both perioperative outcomes and postoperative renal function [7]. Numerous strategies and emerging technologies have been proposed, either individually or in combination [8,9,10]. However, the available evidence remains heterogeneous and inconclusive, resulting in limited and inconsistent guidance within current urological guidelines.

We have developed and refined an approach referred to as no-touch adaptive RAPN [11]. This technique involves a sutureless, off-clamp simple tumour enucleation, with the flexibility to escalate to conventional steps (i.e., arterial clamping, tumour enucleoresection, or renorrhaphy), if deemed necessary. The aim of this strategy is to minimize vascular and parenchymal trauma to the kidney, thereby lowering the risk of perioperative complications and maximizing renal function preservation. The objective of this study was to compare the perioperative outcomes, complications, and impact on short-term renal function of our no-touch adaptive versus conventional technique for RAPN in patients with clinically localized renal tumours.

## 2. Methods

### 2.1. Patients and Study Design

A prospectively maintained database of patients undergoing renal surgery has been active at the Urology Unit of Santa Maria della Misericordia University Hospital, Udine, Italy, since July 2013. The present analysis included all consecutive patients who underwent RAPN for clinically localized T1–T2 renal tumours between June 2017 and February 2026. In June 2023, the surgical team transitioned from the conventional RAPN technique to the no-touch adaptive approach, which was subsequently adopted as the standard technique for all eligible patients. Accordingly, patients treated from June 2023 onward constituted the study group, whereas those treated up to May 2023 served as the historical control group and all received the conventional technique.

This study was conducted in accordance with Good Clinical Practice Guidelines and the Declaration of Helsinki principles. All patients provided a written informed consent and authorized data collection for scientific purposes with anonymous publication. The Institutional Review Board waived the approval of this retrospective study where all patients received a standard-of-care treatment.

### 2.2. Surgical Technique

All procedures were performed by one of five expert surgeons using one of the da Vinci^®^ Si or Xi platforms (Intuitive Surgical, Sunnyvale, CA, USA). The surgical approach (transperitoneal or retroperitoneal) was chosen based on surgeon’s preference. The surgical technique has been described previously [11]. Briefly, the main renal artery is isolated and suspended with a vessel loop. In the study group, no upfront arterial clamping is used. Systemic blood pressure is lowered to a systolic target of 80–90 mmHg. Tumour margins are marked with monopolar cautery, and enucleation is performed with monopolar scissors along an almost avascular plane. Feeding vessels are progressively controlled with monopolar or bipolar coagulation or selectively clipped. If required, intra-abdominal pressure is temporarily increased to 18 mmHg, and suction is used to maintain a clear field. No parenchymal reconstruction is performed. Collecting system defects are selectively closed with 4-0 Monocryl sutures. After tumour excision, haemostasis is checked at low pneumoperitoneum (8 mmHg) and reinforced with coagulation and haemostatic agents, with blood pressure restored to 110–120 mmHg. Arterial clamping and conventional renorrhaphy are reserved for uncontrolled bleeding. In the control group, an on-clamp minimal enucleoresection is performed, followed by renorrhaphy using the sliding-clip technique. The inner layer closes the medulla and collecting system with 3-0 Monocryl, while the outer layer approximates the cortex with 2-0 Vicryl; a single-layer renorrhaphy is used for small defects.

A uniform enhanced recovery pathway was followed throughout the study period. Postoperative analgesia is managed with intravenous non-opioid agents, followed by a progressive switch to oral paracetamol. On POD 1, oral intake is resumed, the urinary catheter is removed, and early ambulation is encouraged. On POD 2, the surgical drain is typically removed, after which patients may be considered for discharge provided urine remains clear and no complications are observed.

### 2.3. Data Extraction and Study Outcomes

Clinical data were retrospectively retrieved from the institutional database by trained medical personnel. Baseline variables included age at surgery, sex, body mass index, Charlson comorbidity index, American Society of Anesthesiologists score, clinical tumour size, clinical tumour stage according to the 8th edition UICC TNM classification, and the Preoperative Aspects and Dimensions Used for an Anatomical (PADUA) classification score [12]. Collected perioperative variables comprised surgical approach, method of arterial inflow management, warm ischemia time (WIT), tumour excision technique, collecting system entry, renorrhaphy strategy, operative time, estimated blood loss (EBL), perioperative blood transfusion rate, and length of hospital stay (LOS). Intraoperative adverse events were classified according to the European Association of Urology intraoperative adverse incident classification system [13]. Procedures in which the no-touch technique could not be entirely accomplished because of the need for arterial clamping, enucleoresection, and/or renorrhaphy were specifically documented and analyzed according to the intention-to-treat principle. Likewise, intraoperative conversions to intracorporeal radical nephrectomy, open radical nephrectomy, or open partial nephrectomy were recorded and included in the intention-to-treat analysis unless otherwise stated.

All surgical specimens underwent standardized pathological processing and were reviewed by a dedicated uropathologist. Histopathological assessment included tumour subtype according to the 5th edition WHO classification, tumour grade based on the 2012 ISUP Consensus Conference grading system [14], pathological tumour stage according to the 8th edition UICC TNM classification, and surgical margin status.

Postoperative complications occurring within 90 days after surgery were prospectively collected and graded according to the Clavien–Dindo classification [15]. If multiple complications per patient occurred, the worst single complication was eventually reported. Complications of grade 1–2 were categorized as minor, whereas grade 3–5 events were considered major complications. Hospital readmissions within 90 days were also registered. Surgical outcomes were reported in accordance with the quality criteria recommended by the European Association of Urology guidelines [16], as summarized in Supplementary Table S1.

Renal function at baseline and at 3 months after surgery was assessed by estimated glomerular filtration rate (eGFR), calculated using the 2021 Chronic Kidney Disease Epidemiology Collaboration equation [17], and categorized according to the 2023 Kidney Disease: Improving Global Outcomes classification [18]. A clinically meaningful deterioration in renal function was defined as a postoperative eGFR reduction of at least 30%, in line with recommendations from a joint workshop of the National Kidney Foundation and the US Food and Drug Administration [19].

Follow-up assessments were generally scheduled at 3 and 12 months during the first postoperative year, every 6 months during the second year, and annually thereafter. Standard follow-up evaluations included physical examination, serum creatinine measurement, and abdominal ultrasonography or computed tomography imaging of the chest and abdomen for up to 5 years after surgery, or earlier if clinically warranted. For the present analysis, only data from the 3-month follow-up visits were included, with patient follow-up censored in June 2026.

Study outcomes were: (1) completion rate of a fully no-touch procedure in the study group and (2) comparative assessment of perioperative outcomes, 90-day postoperative complications, and change in 3-month renal function from baseline in the two groups.

### 2.4. Statistical Analyses

Parametric continuous variables were reported as mean ± standard deviation (SD), whereas median and interquartile range (IQR) were used for non-parametric continuous variables. Student *t* test, Mann–Whitney U test, and Pearson’s chi-squared test or Fisher’s exact test were used to compare continuous parametric, continuous non-parametric and categorical variables, respectively, as appropriate.

As for complications, in addition to between-group grade-by-grade comparisons, we also explored whether some clinical variables, including the type of robotic technique (no-touch adaptive vs. conventional), were associated with any-grade worst single 90-day postoperative complications using an exploratory multivariable logistic regression model. Age and BMI were entered as continuous variables, while sex (male vs. female), ASA score (3 vs. <3), tumour complexity (intermediate/high vs. low PADUA score), and surgical technique (no-touch adaptive vs. conventional) were entered as categorical variables. Regression coefficients, standard errors, odds ratios with 95% confidence intervals (CI), Wald statistics, and *p* values were reported.

As for renal function, both continuous and categorical endpoints were used. Absolute and relative changes in eGFR from baseline to the 3-month follow-up visit were calculated for each patient and compared between groups. To account for baseline renal function, a linear regression model was fitted using 3-month eGFR as the dependent variable, with surgical technique (no-touch adaptive vs. conventional) and baseline eGFR as independent variables. Unstandardized regression coefficients (B), 95% CI, and *p* values were reported for all model terms. Model fit was assessed using the coefficient of determination (R^2^) and adjusted R^2^.

All clinical records were inserted in a dedicated database, and data were analyzed using SPSS v. 21.0 software (IBM Corp., Armonk, NY, USA). All reported *p* values were two-sided, and statistical significance was set at *p* < 0.05.

## 3. Results

The study and control group included 178 and 209 patients, respectively. Demographic, clinical, and pathological variables were all comparable between the two groups (Table 1).

Among the 178 patients allocated to the no-touch adaptive technique, three underwent immediate conversion to intracorporeal radical nephrectomy before any attempt at tumour excision because of high-complexity tumours infiltrating the renal hilum. Therefore, technical completion of the no-touch procedure was assessed in the remaining 175 patients constituting the evaluable intention-to-treat cohort. A fully no-touch procedure was successfully completed in 153/175 patients (87.4%). Reasons for deviation from the planned technique in the remaining 22 patients included conversion to arterial clamping (*n* = 11), renorrhaphy (*n* = 17), enucleoresection (*n* = 6), and conversion to intracorporeal radical nephrectomy for uncontrolled bleeding despite arterial clamping and renorrhaphy (*n* = 1), alone or in combination. Of note, when renorrhaphy was required, it was limited to the bleeding area and consisted of selective sutures only.

Perioperative and postoperative outcomes are detailed in Table 2. No intraoperative complications were observed in either group. Notably, in the study group, WIT was significantly shorter, and fewer cases of collecting system opening were observed. Moreover, LOS was significantly shorter and 90-day readmission rate was significantly lower in the study group.

Any-grade worst single 90-day postoperative complications were significantly less frequent in the study (16.3%) vs. control (40.7%) group (*p* < 0.01). Major complications were also significantly less frequent in the study (3.9%) vs. control (11%) group (*p* = 0.02). Details are reported in Table 3. Notably, no cases of delayed bleeding attributable to intrarenal arteriovenous fistula or renal artery pseudoaneurysm were observed in the study cohort. One incidental renal artery pseudoaneurysm was detected in a patient treated with fully no-touch technique on computed tomography performed on POD 2 following an episode of sudden oxygen desaturation accompanied by a drop in hemoglobin levels in the absence of gross haematuria. The pseudoaneurysm was small (5 mm) and located remotely from the enucleation bed; however, given its association with a large perirenal hematoma, transcatheter arterial embolization was performed.

On exploratory multivariable logistic regression, intermediate/high PADUA tumour complexity was associated with an increased risk of any-grade worst single 90-day postoperative complications (OR 1.25, 95% CI 1.06–1.48; *p* = 0.009), whereas the no-touch adaptive technique was associated with a lower risk (OR 0.35, 95% CI 0.22–0.55; *p* < 0.001). Age, sex, ASA score, and BMI were not significantly associated with the outcome (Table 4).

All 387 patients had available baseline and 3-month eGFR values, and were included in renal function analyses. The median absolute decline in eGFR from baseline to 3 months was significantly smaller in the no-touch group than in the conventional group (−2.1 [IQR −7.5 to 2.8] vs. −7.7 [IQR −14.5 to −0.5] mL/min/1.73 m^2^; *p* < 0.001). Similar findings were observed when eGFR change was expressed as a percentage of baseline (−2.8% [IQR −9.1% to 3.4%] vs. −9.3% [IQR −18.5% to −0.6%]; *p* < 0.001).

In the linear regression analysis adjusting for baseline eGFR, the no-touch adaptive technique was associated with a 5.4 mL/min/1.73 m^2^ higher eGFR at 3 months compared with the conventional technique (B = 5.4, 95% CI 3.6–7.2; *p* < 0.001). Baseline eGFR was also strongly associated with 3-month eGFR (B = 0.92 per mL/min/1.73 m^2^, 95% CI 0.88–0.96; *p* < 0.001), while the model intercept was 1.8 mL/min/1.73 m^2^ (95% CI −1.6 to 5.2; *p* = 0.30). The model showed good explanatory performance (R^2^ = 0.84; adjusted R^2^ = 0.84) (Table 5).

Moreover, the proportion of patients experiencing a clinically meaningful decline in renal function (eGFR reduction ≥ 30%) was significantly lower in the study than in the control group (1.1% vs. 5.7%, *p* = 0.03).

## 4. Discussion

Our study showed that the no-touch adaptive technique for RAPN, consisting of a sutureless, off-clamp simple tumour enucleation with on-demand switch to arterial clamping, tumour enucleoresection, and/or renorrhaphy, was associated with lower morbidity and better short-term renal function preservation compared to the conventional technique.

The rationale underlying the no-touch approach is based on evidence suggesting that both renal ischemia and renorrhaphy may negatively influence postoperative renal function in both the short and long term. In addition, renorrhaphy has been associated with an increased risk of delayed haemorrhagic complications, including pseudoaneurysm formation and arteriovenous fistulas. We further postulated that performing simple enucleation without hilar clamping or parenchymal reconstruction would maximize preservation of the quantity and viability of the residual renal parenchyma, a recognized determinant of long-term functional outcomes following partial nephrectomy [20].

The individual contribution of arterial inflow control, tumour excision, and parenchymal reconstruction to RAPN outcomes remains difficult to define because these technical components are frequently evaluated in combination rather than in isolation. Two recent systematic reviews comparing off- versus on-clamp RAPN found no meaningful differences in most perioperative outcomes, although conflicting results were reported for renal functional preservation and positive surgical margins [21,22]. Likewise, a systematic review of tumour excision techniques suggested that simple enucleation may reduce the need for hilar clamping, shorten hospital stay, decrease complications, and improve postoperative renal function compared with standard resection [23]. Regarding parenchymal reconstruction, most systematic reviews indicate that sutureless techniques may reduce operative time, warm ischaemia duration, and postoperative eGFR decline without increasing blood loss or complication rates, although contradictory findings have also been reported [24,25,26]. Interpretation of these data, however, requires caution. The available literature includes highly heterogeneous patient cohorts with different tumour complexity, baseline renal function, and surgical indications, while surgical techniques vary substantially with respect to clamping strategy, tumour excision, renorrhaphy, haemostatic methods, and surgeon experience. Furthermore, renal functional outcomes are assessed using non-uniform endpoints and follow-up schedules, limiting direct comparisons across studies [27,28]. Accordingly, rather than comparing our results with individual series that differ substantially in these methodological aspects, we have primarily interpreted our findings in the context of the highest available level of evidence, namely systematic reviews and randomized data where available.

Against this background, comparisons between our findings and the published literature should be interpreted in light of the distinctive features of our cohort and, importantly, of our surgical strategy, which combines several technical refinements within a single adaptive framework rather than evaluating one isolated surgical maneuver. Consequently, it is difficult to attribute the observed outcomes to any individual technical component alone.

Our analysis yielded several notable findings. First, a fully no-touch procedure was achieved in the vast majority of patients. In the remaining cases, the adaptive nature of our technique did not preclude the use of conventional maneuvers, including arterial clamping, enucleoresection, and/or renorrhaphy. However, these cases should not be viewed as comparable to those treated with the conventional technique. In fact, when clamping was required, it was only performed for a limited part of the procedure (WIT was significantly shorter), and when renorrhaphy was necessary, only selective sutures (typically one or two) were placed.

Second, in contrast to most reports in the literature, the use of an off-clamp technique under controlled systemic arterial hypotension, combined with progressive coagulation of the resection field and, if necessary, vessel microclipping, resulted in limited EBL (median 80 mL) with a very low perioperative blood transfusion rate (0.6%). These outcomes are comparable to, and in some cases exceed, those reported in contemporary series of on-clamp RAPN, suggesting that meticulous selective monopolar and bipolar coagulation of renal vessels can provide effective haemostasis and challenge long-held surgical paradigms.

Third, no cases of delayed renal bleeding secondary to pseudoaneurysm formation or arteriovenous fistulas were observed. We speculate that this finding may be attributable to the avoidance of routine single- or double-layer renorrhaphy, with only selective parenchymal sutures being placed when deemed necessary. Delayed renal bleeding, although rare, is a potentially life-threatening complication after PN, and requires readmission, as also observed in our series [29]. In these cases, transcatheter arterial embolization is indicated, but carries a risk of renal function decline [30]. The single pseudoaneurysm incidentally observed in our series was considered unlikely to be directly related to the no-touch enucleation technique itself, as it arose remotely from the enucleation bed. Although its exact mechanism cannot be determined retrospectively, it most plausibly resulted from an inadvertent instrument injury during secondary steps of the procedure, or from a pre-existing localized vascular anomaly.

Fourth, avoiding global renal ischemia appears to facilitate recognition of the appropriate dissection plane during simple tumour enucleation. In addition, an incremental haemostasis strategy maintains satisfactory visualization throughout tumour excision while decreasing the likelihood of collecting system violation, which may, in turn, lower the risk of postoperative urinary fistula formation. In the no-touch group, we observed a significantly lower rate of collecting system opening (4.5% vs. 12.4%), although there was no difference in the proportion of cases developing a urinary fistula (1.7% vs. 1.9%).

Fifth, the no-touch adaptive technique was associated with a significantly smaller decline in renal function from baseline to 3 months than the conventional approach. This association remained significant after adjustment for baseline eGFR and was corroborated by a lower proportion of patients experiencing a clinically meaningful (≥30%) decline in renal function. Longer-term follow-up is needed to determine the durability and clinical relevance of the magnitude of the observed difference.

The combination of off-clamp and sutureless RAPN has been only scarcely investigated. The highest level of evidence currently available derives from a recent ad hoc randomized controlled trial including 248 patients, in which Brassetti et al. compared sutureless versus single-layer renorrhaphy off-clamp RAPN performed by a single surgeon [31]. The primary endpoint (trifecta achievement, as previously defined [32]) was comparable between groups (93% in the sutureless group vs. 95% in the renorrhaphy group) and fulfilled the prespecified criterion for non-inferiority. All secondary endpoints (perioperative outcomes) were also similar between the two cohorts. Importantly, all procedures were performed using a purely off-clamp approach, precluding any assessment of the independent impact of renal ischemia. The main technical distinction from our approach concerns the haemostatic strategy: whereas the authors relied on extensive continuous monopolar coagulation of the resection bed to generate a uniform eschar, our technique employs incremental, targeted monopolar and bipolar coagulation throughout tumour excision [33].

We acknowledge the following limitations to our study. First, the retrospective, non-randomized design of this single-centre study should be acknowledged as an inherent limitation. Although the analysis included consecutive patients treated within a prospectively maintained database, residual selection bias and unmeasured confounding cannot be completely excluded. Furthermore, all procedures were performed at a high-volume tertiary referral centre by expert robotic surgeons, which may limit the external validity of our findings. Nonetheless, robotic platforms, perioperative care pathways, and institutional management protocols remained unchanged throughout the study period.

Second, because this study compares two consecutive cohorts treated over different time periods, the possibility of temporal bias cannot be completely excluded. Although all procedures were performed by five experienced robotic surgeons, and therefore the introduction of the no-touch adaptive technique did not coincide with the acquisition of robotic expertise, we acknowledge that progressive improvements in perioperative management or other unmeasured factors may have contributed, at least in part, to the observed outcomes. A potential concern is that the no-touch adaptive technique may entail a higher technical complexity than conventional RAPN, potentially limiting its generalisability. However, this approach does not require the adoption of new instruments or new oncological paradigms. Instead, it reconfigures established RAPN steps into a graded, adaptive workflow, reserving arterial clamping and renorrhaphy for selected cases. By eliminating upfront warm ischaemia, this strategy reduces time pressure during tumour excision and may facilitate recognition of the enucleation plane, thereby potentially shortening the learning curve. Consistent with this hypothesis, a recent two-centre study demonstrated the feasibility and safety of sutureless off-clamp RAPN even when performed by novice robotic surgeons [34]. Nonetheless, formal assessment of learning curves and reproducibility across centres of varying expertise remains warranted.

Third, before implementing this technique on a larger scale, adequately powered, prospective, multicentre, comparative, and possibly randomized studies are required to further investigate its role across the broad range of patient characteristics, nephrometric tumour complexity, and baseline renal function status, and with a reasonably longer follow-up.

## 5. Conclusions

Within the limitations of this retrospective comparative analysis, the no-touch adaptive technique for RAPN was associated with lower perioperative morbidity and better preservation of early postoperative renal function than the conventional approach. This association remained evident after adjustment for baseline renal function and was accompanied by a lower incidence of clinically meaningful renal function deterioration. Although these findings support the rationale for minimizing vascular and parenchymal trauma during tumour excision, adequately powered prospective multicentre studies are required to confirm the reproducibility, durability, and clinical relevance of these functional benefits.

As a final consideration, we strongly believe that future investigations should move beyond the binary comparison between sutureless vs. renorrhaphy-based or on-clamp vs. off-clamp approaches, and instead evaluate individualized adaptive strategies tailored to tumour complexity and intraoperative findings. Such evolution may ultimately better reconcile the competing goals of surgical safety, oncologic efficacy, and maximal renal preservation.

## Figures and Tables

**Table 1:** Demographic, preoperative, and pathological characteristics of the two groups of patients scheduled for robot-assisted partial nephrectomy and included in the comparative analysis.

Variables	Total Cases (*n* = 387)	Study Group (*n* = 178)	Control Group (*n* = 209)	*p* Value
Age (years), median (IQR)	67 (59–73)	68 (60–74)	66 (59–72)	0.36
Male gender, *n* (%)	255 (65.9)	118 (66.3)	137 (65.6)	0.85
BMI (kg/m^2^), median (IQR)	26.3 (24.1–29.8)	25.4 (23.4–29.1)	26.8 (24.6–29.9)	0.51
Charlson comorbidity index > 2, *n* (%)	197 (50.9)	91 (51.1)	106 (50.7)	0.52
ASA score, *n* (%)				0.72
- 1	57 (14.7)	26 (14.6)	31 (14.8)	
- 2	262 (67.7)	120 (67.4)	142 (67.9)	
- 3	68 (17.6)	32 (18.0)	36 (17.3)	
Right side tumour, *n* (%)	191 (49.4)	88 (49.4)	103 (49.3)	0.77
Cystic tumour, *n* (%)	55 (14.2)	26 (14.6)	29 (13.9)	0.55
Clinical tumour size (mm), median (IQR)	35 (27–45)	35 (28–46)	35 (27–45)	0.85
Clinical tumour stage, *n* (%)				0.77
- T1a	292 (75.4)	135 (75.8)	157 (75.2)	
- T1b	89 (23.0)	40 (22.5)	49 (23.4)	
- T2a	6 (1.6)	3 (1.7)	3 (1.4)	
PADUA score, *n* (%)				0.69
- 6–7 (low)	196 (50.6)	91 (51.1)	105 (50.2)	
- 8–9 (intermediate)	152 (39.3)	69 (38.8)	83 (39.7)	
- ≥10 (high)	39 (10.1)	18 (10.1)	21 (10.0)	
Baseline eGFR (mL/min/1.73 m^2^), median (IQR)	79.1 (65.5–102.3)	80.1 (67.3–106.5)	78.4 (65.1–98.3)	0.20
Baseline CKD stage, *n* (%)				0.88
- 1	129 (33.3)	59 (33.1)	70 (33.5)	
- 2	208 (53.7)	96 (53.9)	112 (53.6)	
- 3A	39 (10.1)	18 (10.1)	21 (10.0)	
- 3B	11 (2.8)	5 (2.8)	6 (2.9)	
Tumour histological subtype, *n* (%)				0.54
- Clear cell RCC	182 (47.0)	84 (47.2)	98 (46.9)	
- Non-clear cell RCC	98 (25.3)	48 (27.0)	50 (23.9)	
- Benign	107 (27.6)	46 (25.8)	61 (29.2)	
Pathological tumour stage, *n* (%)				0.35
- T1a	195 (50.4)	94 (52.8)	101 (48.3)	
- T1b	62 (16.0)	28 (15.7)	34 (16.3)	
- T2a	5 (1.3)	2 (1.1)	3 (1.4)	
- T3a	18 (4.7)	8 (4.5)	10 (4.8)	
- NA	107 (27.6)	46 (25.8)	61 (29.2)	
Tumour grade, *n* (%)				0.25
- 1	47 (12.1)	24 (13.5)	23 (11.0)	
- 2	160 (41.3)	76 (42.7)	84 (40.2)	
- 3	31 (8.0)	14 (7.9)	17 (8.1)	
- 4	6 (1.6)	2 (1.1)	4 (1.9)	
- NA	143 (37.0)	62 (34.8)	81 (38.8)	
Positive surgical margins, *n* (%)				0.89
- Negative	372 (96.1)	171 (96.1)	201 (96.2)	
- Positive	11 (2.8)	5 (2.8)	6 (2.9)	
- Indeterminate	4 (1.0)	2 (1.1)	2 (1)	

ASA: American Society of Anesthesiologists; BMI: body mass index; CKD: chronic kidney disease; eGFR: estimated glomerular filtration rate; IQR: interquartile range; NA: not applicable; PADUA: Preoperative Aspects and Dimensions Used for an Anatomical classification; RCC: renal cell carcinoma.

**Table 2:** Perioperative and postoperative outcomes of the two groups of patients scheduled for robot-assisted partial nephrectomy and included in the comparative analysis.

Variables	Total Cases (*n* = 387)	Study Group (*n* = 178)	Control Group (*n* = 209)	*p* Value
Approach, *n* (%)				0.73
- transperitoneal	378 (97.7)	175 (98.3)	203 (97.1)	
- retroperitoneal	9 (2.3)	3 (1.7)	6 (2.9)	
Intraoperative conversion, *n* (%)				0.10
- open partial nephrectomy	3 (0.8)	0 (0)	3 (1.4)	
- robot-assisted radical nephrectomy	9 (2.3)	3 (1.7)	6 (2.9)	
- open radical nephrectomy	2 (0.5)	0 (0)	2 (0.9)	
Arterial inflow control, *n* (%)				<0.01
- on-clamp	198 (51.2)	0 (0)	198 (94.7)	
- off-clamp	164 (42.4)	164 (92.1)	0 (0)	
- off-clamp to on-clamp switch	11 (2.8)	11 (6.2)	0 (0)	
- NA	14 (3.6)	3 (1.7)	11 (5.3)	
Warm ischemia time (min), median (IQR)	14 (10–20)	9 (7–11)	16 (10–23)	0.01
Tumour excision technique, *n* (%)				<0.01
- simple enucleation	174 (45.0)	169 (94.9)	5 (2.4)	
- enucleoresection	199 (51.4)	6 (3.4)	193 (92.3)	
- NA	14 (3.6)	3 (1.7)	11 (5.3)	
Collecting system opening, *n* (%)				0.02
- no	339 (87.6)	167 (93.8)	172 (82.3)	
- yes	34 (8.8)	8 (4.5)	26 (12.4)	
- NA	14 (3.6)	3 (1.7)	11 (5.3)	
Parenchymal reconstruction, *n* (%)				<0.01
- sutureless	158 (40.8)	158 (88.8)	0 (0)	
- selective suturing	16 (4.1)	16 (8.9)	0 (0)	
- single-layer renorrhaphy	20 (5.2)	1 (0.6)	19 (9.1)	
- double-layer renorrhaphy	179 (46.3)	0 (0)	179 (85.6)	
- NA	14 (3.6)	3 (1.7)	11 (5.3)	
Surgical haemostatic agents, *n* (%)				<0.01
- none	34 (8.8)	0 (0)	34 (16.3)	
- Hemopatch^®^	248 (64.1)	166 (93.3)	82 (39.2)	
- Floseal^®^	68 (17.6)	6 (3.4)	62 (29.7)	
- Hemopatch^®^ + Floseal^®^	23 (5.9)	3 (1.7)	20 (9.6)	
- NA	14 (3.6)	3 (1.7)	11 (5.3)	
Operating room time (min), median (IQR)	130 (105–185)	120 (100–175)	130 (105–195)	0.57
Estimated blood loss (mL), median (IQR)	100 (0–300)	80 (0–200)	100 (0–300)	0.55
Perioperative blood transfusions, *n* (%)	7 (1.8)	1 (0.6)	6 (2.9)	0.89
Length of stay (days, median (IQR)	4 (4–7)	3 (3–4)	5 (5–7)	<0.01
90-day readmissions, *n* (%)	9 (2.3)	0 (0)	9 (4.3)	0.01
3-month eGFR (mL/min/1.73 m^2^), median (IQR)	74.1 (59.4–93.3)	79.1 (62.8–99.8)	70.4 (59.7–92.5)	0.03
3-month CKD stage, *n* (%)				0.72
- 1	126 (32.6)	58 (32.6)	68 (32.5)	
- 2	184 (47.5)	86 (48.3)	98 (46.9)	
- 3A	58 (14.9)	27 (15.2)	31 (14.8)	
- 3B	19 (4.9)	7 (3.9)	12 (5.7)	
Absolute ΔeGFR (mL/min/1.73 m^2^), median (IQR)	−4.9 (−11.2 to +0.8)	−2.1 (−7.5 to +2.8)	−7.7 (−14.5 to −0.5)	<0.01
Percentage ΔeGFR (%), median (IQR)	−5.9 (−13.4 to +1.0)	−2.8 (−9.1 to +3.4)	−9.3 (−18.5 to −0.6)	<0.01
3-month eGFR decline ≥ 30%, *n* (%)	14 (3.6)	2 (1.1)	12 (5.7)	0.03

CKD: chronic kidney disease; eGFR: estimated glomerular filtration rate; Δ: change from baseline; IQR: interquartile range; NA: not applicable (intraoperative conversion).

**Table 3:** Worst single 90-day postoperative complications scored according to the Clavien–Dindo classification in the two groups of patients scheduled for robot-assisted partial nephrectomy and included in the comparative analysis.

Grade	Total Cases (*N* = 387), *n* (%)	Study Group (*N* = 178), *n* (%)		Control Group (*N* = 209), *n* (%)		Complication Type	Treatment
1	40 (10.3)	14 (7.9)	7	26 (12.4)	15	Fever of unknown origin	Antipyretics
			2		1	Perirenal hematoma without anemia	Analgesics
			0		1	Hypokalaemia	Potassium supplementation i.v.
			0		1	Pleural effusion	Diuretic
			4		7	Nausea/vomit	Antiemetics
			0		1	Hypotensive crisis	Crystalloids
			1		0	Fall	Analgesics
2	44 (11.4)	8 (4.5)	1	36 (17.2)	3	Anemia	Blood transfusions
			0		2	Perirenal hematoma with anemia	Blood transfusions
			0		1	Arterial hypotension with anemia	Blood transfusions
			1		3	Subcutaneous emphysema	Oxygen
			1		3	Atrial fibrillation	Pharmacological cardioversion
			0		3	Pulmonary embolism	Anticoagulants
			1		1	Agitation/Delirium	Haloperidol
			0		1	Clostridium difficile diarrhea	Antimicrobials
			1		5	Fever of unknown origin	Antimicrobials
			1		2	Pneumonia	Antimicrobials
			1		1	Dyspnoea with desaturation	Oxygen
			1		8	Hypertensive crisis	Antihypertensive drugs
			0		2	Chylorrhoea	Alipidic diet
			0		1	Spontaneous pneumothorax	Oxygen
3a	28 (7.2)	6 (3.4)	3	22 (10.5)	4	Calyceal urinary leakage	Ureteric catheter placement
			1		2	Acute urinary retention	Transurethral catheter placement
			1		0	Acute renal artery thrombosis	Radical nephrectomy
			0		15	AVF/P with gross haematuria	Transcatheter arterial embolization
			1		0	AVF/P without gross haematuria	Transcatheter arterial embolization
			0		1	Epigastric artery bleeding	Transcatheter arterial embolization
4a	2 (0.5)	1 (0.6)	0	1 (0.5)	1	Hypotensive crisis	Intensive cardio-pulmonary support
			1		0	Non-ST-elevation myocardial infarction	Intensive cardio-pulmonary support

AVF/P: arteriovenous fistula/pseudoaneurysm.

**Table 4:** Exploratory multivariable logistic regression analysis of factors associated with any-grade worst single 90-day postoperative complications in the entire cohort of patients scheduled for robot-assisted partial nephrectomy.

Variables	B	SE	Wald χ^2^	OR	95% CI	*p* Value
Age (years), per year increase	0.020	0.013	2.37	1.02	0.99–1.05	0.12
Male (vs. female)	0.010	0.235	<0.01	1.01	0.64–1.60	0.97
ASA score 3 (vs. <3)	0.105	0.320	0.11	1.11	0.59–2.08	0.74
BMI (kg/m^2^), per 1 kg/m^2^ increase	0.049	0.055	0.79	1.05	0.94–1.17	0.37
PADUA score intermediate-high (vs. low)	0.223	0.085	6.87	1.25	1.06–1.48	0.009
No-touch adaptive (vs. conventional) technique	−1.050	0.230	20.83	0.35	0.22–0.55	<0.001

ASA: American Society of Anesthesiologists; B: regression coefficient; BMI: body mass index; CI: confidence interval; OR: odds ratio; PADUA: Preoperative Aspects and Dimensions Used for an Anatomical classification; SE: standard error.

**Table 5:** Linear regression model assessing the association between surgical technique and 3-month eGFR after adjustment for baseline eGFR in the entire cohort of patients scheduled for robot-assisted partial nephrectomy.

Model Term	Unstandardised β Coefficient	95% CI	*p* Value
Intercept	1.8	−1.6 to 5.2	0.30
Baseline eGFR, per 1 mL/min/1.73 m^2^	0.92	0.88 to 0.96	<0.001
No-touch vs. conventional RAPN	5.4	3.6 to 7.2	<0.001

eGFR: estimated glomerular filtration rate; CI: confidence intervals; RAPN: robot-assisted partial nephrectomy.

## Data Availability

The data that support the findings of this study are available from the corresponding author upon reasonable request.

## Supplementary Materials

The following supporting information can be downloaded at https://www.mdpi.com/article/10.3390/jcm15166233/s1, Table S1. Quality criteria for accurate and comprehensive reporting of surgical outcomes recommended by the European Association of Urology Guidelines.
